# Antibiotics-free compounds for managing carbapenem-resistant bacteria; a narrative review

**DOI:** 10.3389/fphar.2024.1467086

**Published:** 2024-09-17

**Authors:** Aref Shariati, Milad Kashi, Zahra Chegini, Seyed Mostafa Hosseini

**Affiliations:** ^1^ Infectious Diseases Research Center (IDRC), Arak University of Medical Sciences, Arak, Iran; ^2^ Student research committee, Arak University of Medical Sciences, Arak, Iran; ^3^ Infectious Disease Research Center, Hamadan University of Medical Sciences, Hamadan, Iran; ^4^ Department of Microbiology, School of Medicine, Hamadan University of Medical Sciences, Hamadan, Iran

**Keywords:** carbapenems-resistant, natural compounds, bacteriophages, nanoparticle, N-acetylcysteine, antimicrobial peptides

## Abstract

Carbapenem-resistant (CR) Gram-negative bacteria have become a significant public health problem in the last decade. In recent years, the prevalence of CR bacteria has increased. The resistance to carbapenems could result from different mechanisms such as loss of porin, penicillin-binding protein alteration, carbapenemase, efflux pump, and biofilm community. Additionally, genetic variations like insertion, deletion, mutation, and post-transcriptional modification of corresponding coding genes could decrease the susceptibility of bacteria to carbapenems. In this regard, scientists are looking for new approaches to inhibit CR bacteria. Using bacteriophages, natural products, nanoparticles, disulfiram, N-acetylcysteine, and antimicrobial peptides showed promising inhibitory effects against CR bacteria. Additionally, the mentioned compounds could destroy the biofilm community of CR bacteria. Using them in combination with conventional antibiotics increases the efficacy of antibiotics, decreases their dosage and toxicity, and resensitizes CR bacteria to antibiotics. Therefore, in the present review article, we have discussed different aspects of non-antibiotic approaches for managing and inhibiting the CR bacteria and various methods and procedures used as an alternative for carbapenems against these bacteria.

## 1 Introduction

The spread of multidrug-resistant (MDR) bacteria is one of the most severe challenges to global health, raising questions about managing these pathogens (Moghadam et al., 2020; Tompkins and van Duin, 2021). Carbapenem resistance in Gram-negative pathogens provides a unique therapeutic problem, as carbapenems have long been regarded as the most productive and strong medicines against MDR Gram-negative pathogens (Doi, 2019). Carbapenems are considered the first line of treatment for infections caused by resistant bacteria, such as *Klebsiella pneumonia, Acinetobacter baumannii, Escherichia coli,* and *Pseudomonas aeruginosa* (Aurilio et al., 2022). Once regarded as the “last resort” antibiotics in many hospitals, this family is a powerful class of broad-spectrum antibiotics that inhibit penicillin-binding proteins, preventing the formation of cell walls (Papp-Wallace et al., 2011). Carbapenem-resistant (CR) bacteria are defined by the United States Centers for Disease Control and Prevention (CDC) as having *in vitro* resistance to at least one carbapenem (Tompkins and van Duin, 2021). Compared to drug-susceptible infections, the higher mortality, hospital stay duration, and CR bacteria expense make them a specific health concern (Zilberberg et al., 2017; Martin et al., 2018). Patients most susceptible to CR bacterial infections include those with underlying conditions and those with indwelling catheters or permanent devices (Voor in ‘t holt et al., 2014; Igbinosa et al., 2020). The carbapenem resistance can be mediated via increases in efflux pump expression, alteration of antibiotic binding targets, decreased membrane permeability and mutation or deletion of pore proteins, and finally, carbapenemase enzymes (Ruppé et al., 2015; Tompkins and van Duin, 2021). Carbapenemases are a diverse family of β-lactamases that have the power to hydrolyze and inactive several antibiotics, including carbapenems, cephalosporins, penicillins, and monobactam. (Tompkins and van Duin, 2021).

The conventional antibiotics that still have anti-CR activity, novel β-lactam-β-lactamase inhibitor combinations that have recently entered the market, and novel aminoglycosides, tetracyclines, and cephalosporins are just a few of the treatment classes that are currently available to treat CR infections (Tompkins and van Duin, 2021). Additionally, combinations of CR-active antibiotics with antibiotics with different mechanisms of action or with “repurposed” medicines from other classes have also shown some promise in treating CR infections (Peyclit et al., 2019). For instance, *in vitro* research demonstrates the effectiveness of mixing colistin with other antibiotics like clarithromycin, rifamycin, or the HIV medication azidothymidine to treat CR bacteria that are also colistin resistant (MacNair et al., 2018). Azidothymidine with tigecycline, pentamidine combined with tigecycline, rifampicin, amikacin, or tobramycin, and polymyxin B combined with sertraline, citalopram, or spironolactone are further combinations that have demonstrated *in vitro* efficacy against CR bacteria (Cebrero-Cangueiro et al., 2018). However, additional animal studies and clinical trials are required to determine the exact efficacy of these combination treatments in actual clinical infections; consequently, the utility of these combination regimens is currently speculative.

Therefore, antibiotic-based approaches were used in recently published studies to inhibit CR bacteria. However, antibiotics could lead to fatal side effects in patients. For instance, an inherent drawback of the extensive utilization of colistin is the elevated prevalence of toxicity, including renal and neurotoxicity, neuromuscular blockade, and occasionally fatal outcomes (Bialvaei and Samadi Kafil, 2015). Additionally, studies reported the quick spread of resistance to the newer CR-active antibiotics. For instance, a recently published study reported that while the general susceptibility to ceftazidime-avibactam is strong, there have been seen mutations that lead to resistance, particularly in bacteria carrying *Klebsiella pneumoniae* carbapenemase (KPC)-2 and KPC-3 enzymes (Hernández-García et al., 2021). To this end, researchers have considered non-antibiotic approaches, such as bacteriophages, natural products and compounds, nanoparticles, etc., for managing CR bacteria. In this regard, this review study aims to examine and discuss the use of the mentioned alternative solutions for inhibiting CR bacteria and destroying their biofilm community.

## 2 Phage therapy

Bacteriophages, or phages, are viruses that can kill bacteria without hurting eukaryotic cells. How bacteria become resistant to phages differs from how they become resistant to antibiotics. Because of this, phages have been used to treat MDR bacteria. Additionally, phage-antibiotic combination therapy may make antibiotic-resistant bacteria susceptible again to conventional antibiotics (Hagens et al., 2006; Chan et al., 2016). Bacteria could be killed more efficiently by a phage cocktail, mixing two or more phages with different host ranges in a single culture (Gu et al., 2012; Jaiswal et al., 2013). Phage cocktails may result in a more effective reduction in bacterial density and an improvement in the activities of the phages. From this perspective, earlier research has demonstrated that phage cocktails significantly reduce bacterial infections (Hall et al., 2012).

Recently published *in vitro* studies reported promising inhibitory effects for phage against CR Gram-negative bacteria, especially *K. pneumoniae*, A. *baumannii*, and *P. aeruginosa* (Table 1). Notably, phages have unwanted traits, including harboring drug resistance and virulence genes that limit their therapeutic application. In this regard, whole-genome sequencing is required to assess the phage genome for antibiotic resistance, toxin, virulence-associated genes, or lysogen-forming gene clusters. Furthermore, phage activity and stability are significantly influenced by the temperature at which they are stored. As a result, different pH and temperature ranges should be used to investigate the inhibitory action of phages. Moreover, phages’ antibacterial effectiveness in clinical situations is enhanced by their high adsorption rate and huge burst size (Mahichi et al., 2009; Li et al., 2020). Collectively, as mentioned, in addition to genome analysis, different characteristics, such as strong lytic activity, relatively broad host range, and high stability, should be evaluated in *vitro* phage studies.

**TABLE 1:** The activities of phages against carbapenems-resistant bacteria.

Year of publication	Phage	CR-bacteria	Outcome	References
2015	BΦ-R3177	*A. baumannii*	This phage showed high stability and lytic activity against host bacteria	Jeon et al. (2015)
2016	BΦ-C62	*A. baumannii*	This phage managed intranasal bacterial challenge in mice and removed bacteria from the lung after 3 days	Jeon et al. (2016)
2017	ϕBO1E	*K. pneumoniae* clade II lineage of CG258	This phage showed strict specificity for targeted bacteria and protected larvae from death following bacterial infection	D'Andrea et al. (2017)
2018	WCHABP1 and WCHABP12	*A. baumannii*	Phage therapy was effective in treating infections in the *G. mellonella* larvae model	Zhou et al. (2018)
2018	vB_Kpn_F48	*K. pneumoniae* Sequence Type 101	This phage showed a short latent period, a narrow host range, and a low burst size	Ciacci et al. (2018)
2019	vB_EaeM_0Eap-3	*Enterobacter aerogenes*	This phage showed an inhibitory effect against 18 of the 28 tested bacteria	Zhao et al. (2019)
2019	Henu1	*K. pneumoniae*	This phage-infected bacteria strains with the capsular types K-1, K-2, and K-57	Teng et al. (2019)
2019	Phage 117 and phage 31	*K. pneumoniae* sequence type 11	The phage cocktail indicated higher antibacterial function than phage 117 alone in LB culture	Tan et al. (2019)
2020	P509	*K. pneumoniae*	This phage at different MOIs decreased the number of bacteria	Li et al. (2020)
2020	vB_KpnP_IME337	*K. pneumoniae*	This phage exhibited an infection lifetime of approximately 90 min, with a latent period of 10 min. Additionally, it showed a high degree of specificity towards the host strain	Gao et al. (2020)
2020	kpssk3	*K. pneumoniae*	This phage was able to lyse 92.59% of clinically isolated bacteria	Shi et al. (2020)
2020	vB_KpnS_Kp13	*K. pneumoniae* K24 capsular type	This phage was effective against all VIM-producing bacteria	Horváth et al. (2020)
2021	BUCT556A	*K. pneumoniae*	This phage showed lytic activity against bacteria	Feng et al. (2021)
2021	TUN1	*K. pneumoniae*	This phage indicated a narrow host range, as it could only lyse K64 *K. pneumoniae* strains	Eckstein et al. (2021)
2022	vB-AbaI-TMU2	*A. baumannii* *P. aeruginosa* *K. pneumoniae*	CR *A. baumannii* was inhibited by phage therapy, while other bacterial strains were resistant to phages	Esmaeili-Fard-Barzegar et al. (2022)
2022	P13	*K. pneumoniae*	This phage indicated a large lytic plaque after overnight coculture with its host bacteria	Fang and Zong (2022)
2022	Eight different phages	*K. pneumoniae*	All of the phages significantly decreased the number of bacteria	Baqer et al. (2022)
2023	Abp95	*A. baumannii*	The phage showed a beneficial effect on wound healing in a diabetic mouse wound infection model by effectively eliminating local infections	Huang et al. (2023a)
2023	vB_PseuP-SA22	*P. aeruginosa*	This phage decreased the number of live bacteria (five logs) in the biofilm community	Teklemariam et al. (2023)
2023	vB_KpnS_SXFY507	*K. pneumoniae*	This phage showed antibacterial activity and increased *Galleria mellonella* larvae survival rate after infection	Feng et al. (2023)
2023	vB_KshKPC-M	*K. pneumoniae*	This phage showed high killing activity against planktonic and biofilm forms of bacteria	Mohammadi et al. (2023)

CR: carbapenems-resistant. MOI: Multiplicity of infection. VIM: Verona integron-encoded metallo-β-lactamase. LB: luria broth.

Because the biofilm community is one of the most significant problems in treating CR bacterial infection, phage interactions with biofilm are an essential topic in *vitro* investigation. Due to the inability of antibiotics to penetrate the complex polysaccharide matrix (glycocalyx) of biofilms, they are 10–1,000 times more resistant to antibiotics than planktonic organisms. To that purpose, recent research found that phages could limit biofilm formation and remove the mature biofilm of CR bacteria (Wu et al., 2019; Santiago et al., 2020; Mulani et al., 2022). Notably, phages can enter the biofilm and obliterate its structure by triggering the production of enzymes such as polysaccharide depolymerase. Besides, this enzyme can specifically destroy the host bacterial envelope’s macromolecule carbohydrates (Yan et al., 2014). The findings of the experiments showed that natural lytic phage can diminish the biofilm community of CR bacteria by phage-induced lysis and exopolysaccharide degradation (Vukotic et al., 2020; Hao et al., 2021; Li J. et al., 2021; Li M. et al., 2021).

Depolymerase pretreatment of the biofilm followed by other antibacterial agents may be a viable alternative for managing bacterial biofilm. In a study, the scientists managed the MDR *K. pneumonia* biofilm using ciprofloxacin, recombinant phage-encoded enzyme, and lytic phages (producing and non-producing depolymerase). The results demonstrated that ciprofloxacin and depolymerase-producing phage were the most effective antibiofilm combination against bacterial biofilm (Latka and Drulis-Kawa, 2020). Additionally, *Wu* et al. reported that depolymerase could enhance the polymixin activity against *K. pneumoniae* biofilms when combined with antibiotics (Wu et al., 2019). Besides, another experiment showed that capsule depolymerase could make CR *K. pneumoniae* fully susceptible to the killing effect of serum complement (Liu Y. et al., 2020). Therefore, phage depolymerase should be considered for managing CR *K. pneumoniae* biofilm; however, more confirmatory studies are required.

The combination of phages with antibiotics was also used to manage CR bacteria. For instance, in 2022, a study’s findings demonstrated that combining a two-phage cocktail and imipenem effectively delayed carbapenemase growth-producing *K. pneumoniae* (Michodigni et al., 2022). Another study reported that combined usage of gentamycin and phage treated the mice with acute pneumonia caused by MDR *K. pneumoniae* (Wang et al., 2021). Consistent with these findings, using phage in conjunction with colistin was more effective at preventing the growth of CR *A. baumannii* than either treatment alone (Wintachai et al., 2022).

Although the precise mechanism of the interaction between phages and antibiotics has not yet been determined, recent investigations have indicated various potential pathways. The sensitivity of the chosen antibiotic to the particular bacterial strains following phage activity may cause the synergistic activity between antibiotics and phage cocktails. Phage-resistant bacterial strains are more vulnerable to antibiotics and develop more slowly than wild ones. Phages particularly alter the bacterial surface structures (outer membrane proteins, polysaccharides, etc.), removing obstacles to the entry of various antibiotics, demonstrating that phages have the impact of increasing bacterial antibiotic sensitivity (Wang et al., 2021; Michodigni et al., 2022; Wintachai et al., 2022). Notably, during the last stages of the replication cycle, phages produce endolysins to breach the bacterial cell wall and produce offspring virion. Endolysins demonstrated effective inhibition of Gram-positive bacteria, but their ability to inhibit Gram-negative bacteria was constrained by the existence of the outer membrane (Schmelcher et al., 2012; Baliga et al., 2022). Colistin can enhance endolysins’ capacity to overcome the outer membrane’s impermeability (Baliga et al., 2022).

Phage-encoded depolymerases play a role in the degradation of the host bacterium’s EPS, LPS, and capsular polysaccharides during phage invasion. Hence, phages can dismantle the biofilm architecture and enhance the infiltration of antibiotics into the inner layers of the biofilm by stimulating the production of enzymes like polysaccharide depolymerase. Therefore, antibiotic-phage combination therapy shows potential as a treatment strategy for controlling CR bacteria, including their biofilm population (Hanlon, 2007).

As mentioned in the previous paragraphs, *in vitro* studies reported different phages for managing CR bacteria. Moreover, other studies evaluated the function of phages against CR bacteria in animal studies and clinical seating. In this regard, intraperitoneally injection of phages, controlled *K. pneumoniae* infection in mice. Another study also showed that phages distributed more rapidly into the systemic circulation via the intraperitoneal route than the oral route (Dhungana et al., 2021; Shi et al., 2021; Bai et al., 2022; Li et al., 2022b)*. Liang* et al. reported phage therapy leads to a better survival rate in mice with CR *K. pneumoniae* bacteremia than ceftazidime/avibactam and tigecycline (Liang et al., 2023). Another investigation used intra-rectal and oral therapy with a custom-made phage to treat patients with multi-site colonization of CR *K. pneumoniae* (Corbellino et al., 2020). In addition to *K. pneumoniae*, phage therapy was used to manage CR *A. baumannii* and *P. aeruginosa* infection in animal models and clinical settings. To this end, phage therapy successfully manages CR *A. baumannii* acute pneumonia and lung infection in mice (Hua et al., 2017; Jeon et al., 2019).

Furthermore, phage therapy indicated promising results in treating patients with CR *A. baumannii* lung infection (Tan et al., 2021; Wu et al., 2021). Finally, two-phage cocktails formulated as hydrogels inhibited CR *P. aeruginosa* wound infection in animal models. Combined phages and conventional antibiotics successfully managed patients with empyema caused by this bacterium (Chen et al., 2022).

Therefore, in addition to *in vitro* studies, animal models and preclinical studies also reported promising effects for phage therapy against CR bacteria. Nevertheless, phages still possess constraints in their actual implementation in clinical settings. Phage formulations exhibit varying *in vivo* pharmacokinetics and pharmacodynamics compared to antibiotic therapy. Since the preparations of different phages have distinct biological characteristics, there are significant variations in actual clinical applications. There is a lack of established guidelines regarding the optimal dosage, duration, and method of administering phage therapy, and there are no conventional treatment protocols for phage therapy. Furthermore, when phage preparations are made, specific endotoxins are created that could be cytotoxic and immunogenic. In the end, certain bacteria have acquired resistance to phage infection through various mechanisms, including adsorption resistance, spontaneous mutations, receptor and penetration blocking systems, and adaptive immunity linked to CRISPR/Cas systems (Hibstu et al., 2022). Hence, although phage therapy showed promising effects for managing CR bacteria, the mentioned issues should be evaluated in future studies.

## 3 Natural products

Research has demonstrated that plant-derived substances, such as essential oils (EOs), extracts, and pure chemicals, substantially impact bacteria and their biofilm community. The bioactive constituents derived from various plant components, including roots, leaves, and fruits, possess therapeutic characteristics and exhibit distinct medicinal effects upon modification (Ahmad et al., 2015; Rangel et al., 2018). To this end, recently published studies used different natural products to inhibit CR bacteria and their biofilm community. The exact interaction of natural products and CR bacteria is not elucidated yet, but in this section, we will discuss some of the most important antibacterial mechanisms of natural products.

According to research, eugenol, a phenolic aromatic compound primarily derived from cinnamomum and clove essential oils, destroys the membrane integrity of CR *K. pneumoniae* by producing reactive oxygen species (ROS), and glutathione depletion, causes the leakage of bacterial cytoplasmic components like protein, β-galactosidase, and DNA. Moreover, when eugenol comes into contact with bacterial biofilm, the entire matrix’s thickness diminishes, and its integrity is lost (Liu et al., 2023). Another investigation also reported that the cell membrane of CR *K. pneumoniae* was harmed by eugenol, as indicated by a drop in intracellular ATP concentration, a reduction in intracellular pH, cell membrane hyperpolarization, and an increase in membrane permeability. In addition, eugenol disrupted the cellular structure and caused the loss of internal components in CR *K. pneumoniae* (Qian et al., 2020).

Thymol and carvacrol, the main ingredients of different EOs of various aromatic plants, also showed promising inhibitory effects against CR bacteria. In this regard, the findings of the studies showed that the lipophilic nature of these compounds and their accumulation in cell membranes are related to their antibacterial properties. This interaction inhibits electron transport for energy production and disrupts the proton motive force, synthesis of cellular components, and protein translocation. Cell lysis and death may occur as a result of these physiological changes. Lipopolysaccharide (LPS), a powerful barrier for hydrophobic compounds, including hydrophobic antibiotics, is found in the outer membrane of Gram-negative bacteria. Thymol and carvacrol, lipid-based compounds made from γ-terpinene, can assist in transporting hydrophobic antibiotics inside cells (Nazzaro et al., 2013; Kwiatkowski et al., 2022). In line with these results, *De Souza* et al., after observation of carvacrol inhibitory effect against carbapenemase (KPC)-producing *K. pneumoniae*, supposed that the inhibitory effects of carvacrol can be attributed to its interactions with the structural and functional properties of the cytoplasmic membrane. Carvacrol interacts with the lipid bilayer and positions itself between fatty acid chains, causing the expansion and destabilization of the cytoplasmic membrane (de Souza et al., 2021). Additionally, carvacrol and other hydrophobic substances may enter the bacterial cell’s outer membrane pores and the periplasmic region. Carvacrol fits between fatty acid chains because it can bind to hydrogen, letting ions leave the cytoplasm. Cell membrane destabilization makes membranes more fluid and cells more permeable (Amaral et al., 2020).

Phytol, a diterpenes alcohol from chlorophyll widely used as a food additive and in medicinal fields, is also reported as the antibiofilm agent, inhibiting exopolysaccharide production as well as initial cell attachment, hypermucoviscosity, and curli expression in CR *K. pneumoniae* (Adeosun et al., 2022b). In another experiment, the authors used linalool to inhibit this bacterium. The results demonstrated a notable decrease in the quantity of cytoplasmic and membrane proteins, suggesting that the cells of KPC- *K. pneumoniae* treated with linalool had damage to their membranes. The presence of oxidative stress was confirmed by the downregulation of proteins sensitive to oxidative stress and the overexpression of proteins that regulate oxidative stress. The zeta potential measurement and outer membrane permeability assay demonstrated that linalool enhances the bacterial surface charge and the membrane’s permeability. Linalool therapy detected intracellular leakage of nucleic acid and proteins (Yang et al., 2021).

Finally, *Yang* et al. reported that cinnamomum has antimicrobial effects on KPC- *K. pneumoniae* cells by disrupting their cell membranes. Proteomic profiling reveals that the membrane damage is caused by oxidative stress. Cinnamomum treatment disrupted the production process of the plasma membrane, cell wall, and outer membrane, impairing the structural repair system. The oxidation due to this process damages the bacterial membrane, eventually allowing ROS to enter the cells. Simultaneously, it also causes the leakage of intracellular contents. ROS causes genetic damage and hinders the functioning of DNA and membrane repair mechanisms (Yang S. K. et al., 2019). Recent investigations have collectively demonstrated that natural products induce oxidative stress, damage bacterial membranes, cause cellular leakage, and result in cell death.

It is noteworthy to mention that new antimicrobial agents are required to reduce the toxicity of conventional antibiotics. Moreover, combination therapy could enhance the efficacy of different antibiotics (Pinto et al., 2009; Ahmad et al., 2010). In this concept, the combined use of natural products and other antibiotics was considered to inhibit CR bacteria and their biofilm community. To this end, *Yadav* et al. reported that a water-soluble curcumin derivative inhibited the AcrAB-TolC efflux system in MDR *K. pneumoniae* by disrupting the membrane potential and causing depolarization. Combining this compound and meropenem was highly synergistic, reducing drug dose regimes and toxicity (Yadav et al., 2021). In line with these results, a study published in 2020 also reported that a combination of colistin + curcumin showed a remarkable reversal in colistin minimum inhibitory concentration (MIC) in Enterobacteriaceae. Noteworthy, the authors proposed that efflux inhibition is the primary mechanism responsible for curcumin’s synergistic and modulation ability (Sundaramoorthy et al., 2020). It can be concluded that curcumin can shut down the efflux system because almost all efflux pump systems need energy (ATP) for their functions, and the curcumin that inhibits the proton motive force will inhibit ATP generation. Therefore, this natural compound could improve the activity of antibiotics by targeting efflux pumps.

In another investigation, the researchers reinstate the efficacy of carbapenem against CR *K. pneumoniae* by employing celastrol and thymol. The results of this study indicate that celastrol alone did not have an impact on meropenem-MIC, and when combined with thymol, it only caused a 2-fold drop. However, both celastrol and thymol resulted in a significant decrease of 4–64 folds in meropenem-MIC. Celastrol effectively inhibited carbapenemase activity, but its access to the target was hindered by the outer membranes of Gram-negative bacteria, which act as a barrier to its penetration. However, thymol exerts its effects by disrupting the outer membranes and porins through its lipophilic action. It does this by integrating into the polar head groups of the lipid bilayer, which leads to changes in the permeability of the cell membrane. In summary, thymol enhances the capacity of celastrol and meropenem to pass through the CR *K. pneumoniae*. Additionally, celastrol suppresses carbapenemase-hydrolytic activity. Hence, thymol-meropenem-celastrol combination therapy can efficiently kill CR bacteria (Abdel-Halim et al., 2022).

Furthermore, a recently published study reported synergistic effects of polymyxin B in combination with *Cinnamomum cassia* L. EO (CEO) against carbapenemase-producing *Serratia marcescens* and *K. pneumoniae*. Notably, the CEO successfully suppressed the germs stated and achieved this suppression by combining with polymyxin B at a lower dosage of antibiotics. It is possible to assume that CEO caused damage to cell membranes, and this damage destabilized the outer membrane of the carbapenemase-producing bacteria, which then allowed polymyxin B to enter the periplasm of the cell. As a result, the outer membrane loses its integrity, leading to the leakage of cellular contents and ultimately causing cell death (Vasconcelos et al., 2020).

In the end, fisetin (1; 3,7,3′,4′-tetrahydroxyflavone) is a type of flavonoid that has been shown to have anticancer, antiangiogenic, antiviral, anti-invasive, and anti-aging effects. These features are due to its property of creating free radicals (Jash and Mondal, 2014). Until now, various experiments have been conducted to measure the effectiveness of fisetin in treating diseases caused by bacterial pathogens. The studies showed that fisetin has activities against the lipopolysaccharide of Gram-negative bacteria and can reduce the activity of pathogens in human cells. In addition, fisetin significantly suppressed the expression of nitric oxide (NO), prostaglandin E2 (PGE2), and cytokines, such as interleukin-6 (IL-6) and tumor necrosis factor-alpha (TNF-), which are pro-inflammatory factors. Fisetin can also inhibit the biofilm formation of bacterial cells such as MDR *A*. *baumannii*; however, the exact interaction of fisetin and the biofilm community of this bacterium was not reported (Raorane et al., 2019).

Studies reported that fisetin inhibits CR *K. pneumoniae* (Adeosun et al., 2022a; Zhang et al., 2022c). One of the main classes of beta-lactamases is OXA-48, which was identified in Turkey in 2001 and spread worldwide. This enzyme makes the organism resistant to carbapenems and penicillins but cannot hydrolyze cephalosporins (Poirel et al., 2004). A study showed that fisetin effectively restored the antibacterial efficacy of piperacillin or imipenem against *E. coli* producing OXA-48, resulting in a 2–8-fold reduction in minimum inhibitory concentration (MIC) (Zhang et al., 2022b). In line with these findings, *Adeosun* et al. introduced fisetin as the best anti-CR K*. pneumoniae*, demonstrating a MIC value of 0.0625 mg/mL. This compound inhibited curli expression, a type of fimbriae composed of proteins called curlins and functional amyloid surface fiber, and reduced hypermucoviscosity (Adeosun et al., 2022a). Finally, a recently published study evaluated fisetin’s effect on NDM-producing *E. coli*. Molecular dynamics simulations revealed that fisetin successfully inhibits the hydrolytic activity of NDM-1. Notably, the mutation of NDM-1 resulted in a decreased inhibition of NDM-1 activity by fisetin compared with the wild-type protein. To this end, the authors proposed that fisetin is an effective NDM-1 inhibitor, which suggests the combination of this compound with meropenem is a promising strategy for CR bacterial infection (Guo et al., 2022). Therefore, fisetin can be employed as a model in the search for new medications or as an alternative in regulating the pathogenicity of CR *K. pneumoniae*.

**FIGURE 1:**
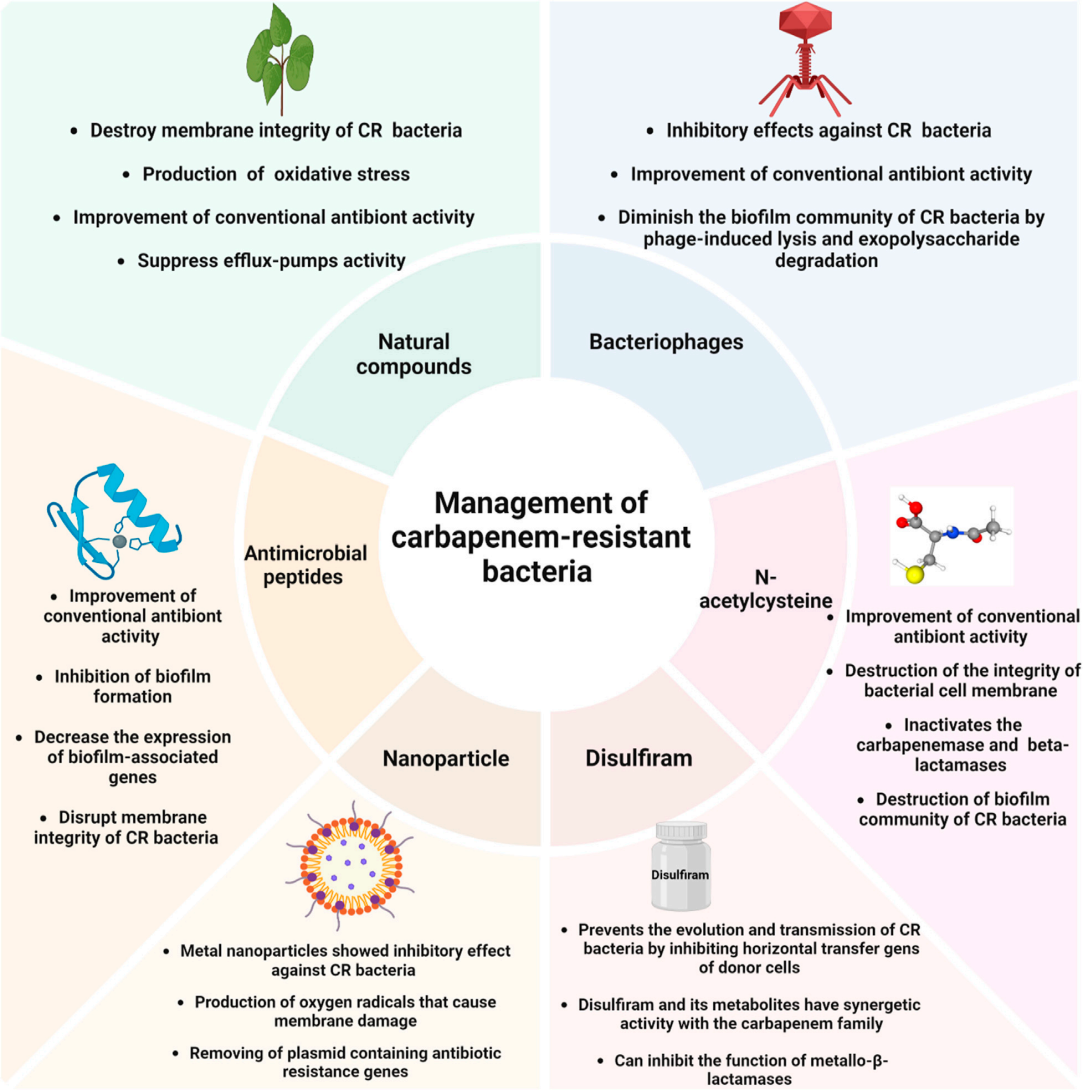
Non-antibiotic approaches for managing carbapenem-resistant bacteria.

Therefore, as mentioned, natural compounds could inhibit CR bacteria and enhance the performance of antibiotics against these bacteria (Figure 1). Nevertheless, the effectiveness of natural compounds is frequently impeded by their low solubility in water, tendency to evaporate, and vulnerability to degradation by light and oxidative substances. The use of different drug platforms may resolve these limitations. For instance, *Tayeb* et al. found that the developed nanoemulsion significantly improved the antibacterial effectiveness of meropenem and clove EO. This nanoemulsion showed promise as a carrier for antimicrobial substances (Tayeb et al., 2022). Besides, chitosan-coated nanoemulsion showed potential and effective intranasal formulation against CR *A. baumannii* and *K. pneumoniae* (Rinaldi et al., 2020). Therefore, due to their strong physical and chemical properties and ability to kill bacteria, drug delivery systems have the potential to enhance treatment choices for human infections and serve as more effective carriers for drugs with limited bioavailability. Implementing this approach might mitigate drug toxicity and prolong the efficacy of antibacterial treatments that are already on the market. Due to their high cellular absorption and controlled release distribution, nanostructured devices have been devised to encapsulate EO to increase their bioavailability and bioefficacy. However, data about nano-platform usage for enhancing the efficacy of natural products against CR bacteria is limited, and more confirmatory studies are needed in this field.

## 4 Disulfiram

Disulfiram (Tetraethylthiuram disulfide), primarily known as “Antabuse,” was first introduced in 1981 as a drug for the treatment of chronic alcoholism patients by inhibiting the function of erythrocyte aldehyde-dehydrogenase enzyme by producing acute sensitivity to alcohol (Kleczkowska et al., 2021). Later, it was introduced to have toxic activity against lower forms of life utilizing copper-containing respiratory enzymes and have inhibitory function against copper-chelating enzymes, especially in fungal and bacterial cells (Tisato et al., 2010; Dubey et al., 2022). The antibacterial activity of disulfiram and its derivatives has been proven in recent years. For example, *Thakare* et al. reported the antibacterial activity of disulfiram, which could successfully cause the disappearance of *Staphylococcus aureus* biofilm and its intracellular population (Thakare et al., 2019).

In addition, disulfiram prevents the evolution and transmission of CR bacteria by inhibiting horizontal transfer genes of donor cells through plasmid to another cell and minimizing the spread of meropenem-resistant cells. Moreover, disulfiram and its metabolites have synergetic activity with the carbapenem family, such as meropenem, and have a destructive effect on the biofilm formation of CR bacteria. In one study conducted on CR bacteria, it was shown that not only disulfiram could enhance the antibacterial activity of meropenem and colistin against bacteria but also increase the potent ability of colistin by damaging bacterial cell membranes (Chen et al., 2023). A study by *Dubey* et al. aimed to investigate the effect of meropenem in combination with disulfiram against CR *A*. *baumannii* infections. The results reported that disulfiram has a successful synergetic effect on New-Delhi metallo beta-lactamase (NDM) and IMP-type metallo-β-lactamases. The mechanism is assumed that disulfiram binds to NDM and chelates the Zink active site. It also makes a disulfide bond through its Cys208 residue, which has an inhibition function in the meropenem hydrolysis process (Dubey et al., 2022). Other similar studies were also done to determine the activity of disulfiram on the inhibition of CR bacteria. The results showed that using disulfiram as an adjuvant increases the drug’s efficacy (Chen et al., 2023). Therefore, as mentioned, disulfiram can inhibit the function of metallo-β-lactamases in CR bacteria and improve the function of carbapenems against these bacteria. However, there is limited data about the interactions of disulfiram and CR bacteria; therefore, this compound should be considered for future studies on managing CR bacteria.

## 5 Metal nanoparticles

Recent advances in nanotechnology have provided the ability to prepare nanoparticles of specific size and shape, which can help develop new antibacterial agents. The advantage of nanomaterials over traditional antibiotics is due to their surface area and chemical reactivity compared to bulk materials (Shariati et al., 2022; Hosseini et al., 2023b; a). Therefore, nanoparticles have attracted a lot of attention in biomedicine. Metal nanoparticles became the main focus of many biomedical applications, including antibacterial agents, due to their tunable shape- and size-dependent properties. Due to the antimicrobial nature of metal nanoparticles such as copper (Cu), silver (Ag), zinc (Zn), iron (Fe), and titanium (Ti), they can be used against MDR bacteria (Huh and Kwon, 2011). The critical point is that biogenic nanoparticles have long-term stability and are biocompatible; therefore, they are used primarily for antimicrobial applications. The mechanisms of antimicrobial action of biogenic nanoparticles include metal ion release, oxidative stress, and non-oxidative stress that can occur simultaneously (Huh and Kwon, 2011; Fernandez-Moure et al., 2017).

The findings showed that combining antibiotics with biogenic metal nanoparticles can be useful for increasing their antimicrobial activity. Additionally, by examining nanoparticles synthesized by biological and chemical methods, it was found that biological nanoparticles have a better antimicrobial effect than nanoparticles synthesized by chemical methods (Singh et al., 2018).

### 5.1 Effect of silver nanoparticles on carbapenem-resistant bacteria

One of the causes of death due to *A. baumannii* is resistance to most of the antibiotics used for treatment. Resistance to carbapenem, the most effective beta-lactam antibiotic for *Acinetobacter*, is a significant challenge and concern. Lung inflammation is one of the important symptoms of pneumonia caused by *A. baumannii*, which leads to the destruction of the epithelial barrier. The interaction between this bacterium and human lung cells (alveolar epithelial) due to its adhesion and invasion of these cells leads to infection and causes cell death (Lee et al., 2001; Choi et al., 2008). One of the reasons for the pathogenicity of *A. baumannii* is its ability to survive in human lung cells. Therefore, investigating this bacterium’s interaction with the host cell can be helpful. Silver nanoparticles (AgNPs) have antibacterial properties against different organisms due to their different mechanisms of antimicrobial action. Polyvinylpyrrolidone (PVP) is a stabilizer with minor sensitivity to pH changes and surface charge changes. Recent reports have shown that AgNPs conjugated with PVP are more stable than other AgNPs; it has also been found that PVP-conjugated AgNPs are less toxic to mammalian cells (Tiwari et al., 2017). Studies have reported that AgNPs conjugated with PVP have better antimicrobial activity *in vivo* than other AgNPs. Hence, they can be used as alternatives to carbapenems (Gnanadhas et al., 2013).

The study of *A. baumannii* infection in the human lung host cell (A-549) is a good model for surface interaction with bacteria. In a study, AgNPs conjugated with PVP were synthesized using chemical methods, and their effectiveness against CR *A. baumannii* was investigated (Tiwari et al., 2012). The survey of this cell type showed that during bacterial infection, about 40% of the bacteria adhered to the A-549 cell line, while 20% entered the cell, causing a threefold increase in the production of ROS. In this regard, 30 μM PVP-AgNPs showed antibacterial activity against CR *A. baumannii* strain, and this concentration had no cytotoxic effect on the human lung cell line. Noteworthy, the results of this study showed that during A*. baumannii* infection, ROS concentrations increased up to threefold. PVP-AgNP treatment causes a decrease of about 80% in the viability of intracellular bacteria (Tiwari et al., 2012). Therefore, it can be concluded that AgNPs conjugated with PVP can be a suitable alternative to the current antibiotics used against CR *A. baumannii*.

Metals and their alloys, synthetic and natural polymers, have clear characteristics that make them candidates for biomedical applications. Among these metal nanoparticles, metal oxides, such as zinc oxide (ZnO), have attracted much attention today because they can be stable at low temperatures and in various conditions (Tiwari et al., 2018). One of the remarkable points of ZnO particles is that they have antibacterial activity against Gram-negative and Gram-positive bacteria and also activity against bacterial spores (Hoseinzadeh et al., 2014). It has been pointed out that ZnONPs are low toxicity, biocompatible, and bio-safe. Considering that the mechanism of antimicrobial activity of ZnONPs has not been well explained, several studies have suggested that the production of hydrogen peroxide can be one of the main factors of its antimicrobial activity, and also, the binding of ZnONPs on the surface of bacteria can have an inhibitory effect (Hoseinzadeh et al., 2014). To this end, *Vishvanath* et al. have investigated the antimicrobial activity of ZnONPs against CR *A. baumannii* (Tiwari et al., 2018).

ZnONPs can be used as an alternative to carbapenem antibiotics that inhibit the growth of CR *A. baumannii* by producing oxygen radicals that cause membrane damage. Therefore, ZnONPs can be considered an alternative carbapenem antibiotic drug against CR *A*. *baumannii*. The virulence of *A. baumannii* is influenced by its ability to survive in human lung cells; therefore, it is crucial to study the effect of zinc oxide in the interaction of *A. baumannii* with human lung host cells. Targeted delivery of nanoparticles to lung cells in an animal model requires further studies to make it a suitable drug against *A. baumannii*. Also, to identify proteins involved in the mechanism of action of this nanoparticle, an accurate proteomic analysis of *A. baumannii* in the presence of ZnO is needed (Tiwari et al., 2018).

Drug resistance traits are rapidly spread among bacteria by horizontal gene transfer, especially through plasmids. Pectin-coated platinum nanoparticles (ptNps) at a concentration of less than 20 μM are effective in removing the plasmid containing extended-spectrum beta-lactamase (ESBL) in *E. coli* (Bharathan et al., 2019). Plasmid curing means plasmid loss from bacterial strain due to treatment with different compounds. Plasmid removal from the host mainly occurs by two mechanisms: 1) inhibition of plasmid replication and 2) interfering with plasmid segregation. So far, many plasmid curing agents are known, including ethidium bromide surfactants such as SDS, glycine, organic heterocyclic compounds, acridine orange, and specific plant metabolites, such as plumbagin. Most of these factors were only in the environment. Either they were toxic *in vivo*, or their efficacy as *in vivo* plasmid curing agents has not been investigated previously (Molnar et al., 1977; Crameri et al., 1986; Spengler et al., 2006).

*Bharathan* et al. developed pectin-capped platinum nanoparticles (PtNPs) to treat fish infected with CR bacteria and rescue fish from infection without additional toxicity (Bharathan et al., 2019). PtNPs controlled the infection in fish and enhanced the adaptive immune response against pathogen re-entry; thus, the fish survived repeated infections. PtNPs can act as a plasmid removal agent in the clinical isolate of MDR *E. coli* in a fish infection model. Investigations by PCR method showed that the plasmid contains bla-_OXA23_, bla_NDM-5,_ and bla OXA-48 genes that can encode carbapenem resistance. Various techniques are used to investigate the plasmid curing mechanism, such as membrane permeability, TEM imaging, ROS production, and membrane potential integrity (Buckner et al., 2018). It shows that sub-MIC levels of PtNPs interact with the cell surface and compromise the integrity of the inner membrane. Gyrase inhibition assay showed that treatment of bacteria with PtNP in the presence and absence of gyrase caused DNA cleavage even at concentrations lower than the MIC, which may account for the ability of PtNPs to eliminate plasmid (Bharathan et al., 2019).

PtNPs at a concentration of less than 20 µM caused the formation of colonies with small morphology and decreased the growth of CR in laboratory conditions and the body. The treated strain (lacking plasmid) had less resistance to meropenem and ceftriaxone than the wild type. Also, the treated strain showed a 50% reduction in biofilm formation compared to the wild type. This study demonstrated for the first time that Biogen’s PtNPs induced selective plasmid loss from *E. coli* strain U3790, leading to a significant decrease in MICs for meropenem and ceftriaxone. The absence of the plasmid leads to the formation of small colony variants, which have slower growth. Importantly, this study showed that nanoparticles are non-toxic and can cause plasmid loss *in vivo*. Its combined administration with meropenem causes a significant reduction in bacterial load compared to treatment with meropenem alone (Bharathan et al., 2019).

## 6 N-acetyl cysteine

N-acetyl cysteine (NAC) is the N-acetyl form of the amino acid L-cysteine. While it is not classified as an antibiotic, it does have antibacterial capabilities and the ability to destroy biofilms. Additionally, it has shown promise in removing bacteria already attached to stainless steel surfaces (Olofsson et al., 2003; Zhao and Liu, 2010; Wendorf et al., 2015). Different studies have demonstrated the *in vitro* efficacy of NAC in inhibiting the growth of bacteria and preventing the formation of biofilms. These effects have been observed in Gram-positive and Gram-negative microorganisms, including *Burkholderia cepacia complex, Stenotrophomonas maltophilia,* and *P*. *aeruginosa* (Parry and Neu, 1977; Roberts and Cole, 1981; Alfredsson et al., 1987; Blasi et al., 2016; Pollini et al., 2018b; Aiyer et al., 2021; Alarfaj et al., 2022).

NAC possesses antioxidant and anti-inflammatory characteristics by enhancing glutathione production, which helps neutralize harmful oxygen radicals and counteract the effects of proinflammatory cytokines (Zafarullah et al., 2003; Tenório et al., 2021). The NAC counteracts the harmful effects of free radicals, diminishes oxidative stress and inflammation, and enhances the functioning of the immune system. In addition, NAC exhibits vasodilatory effects on microcirculation, leading to an improvement in locoregional blood flow (Forman et al., 2009; Chertoff, 2018). The events mentioned above can have significant ramifications in an unregulated host response to infection, characterized by a high release of pro-inflammatory cytokines, ROS, and profound disruption of microcirculation, as observed in septic shock (Ince, 2005; Ait-Oufella et al., 2010; Angus and Poll, 2013; Ince et al., 2016). In this regard, animal studies have shown that NAC improves organ damage caused by endotoxin shock by reducing the formation of free radicals and inflammatory cytokines (Hsu et al., 2006). Therefore, NAC can serve as a valuable adjunctive therapy in infectious disorders, mitigating organ damage and protecting against septic shock. Given that septic shock is distinguished by excessive and unbalanced production of pro-inflammatory cytokines, ROS, and significant disruption of circulation, the use of substances that can counteract these effects is justified in treating this illness (Oliva et al., 2021).

As mentioned, NAC is a substance that has antioxidant and anti-inflammatory properties. It can be utilized alongside antimicrobial therapy to treat severe infections caused by MDR organisms, such as CR *K. pneumoniae* and CR *A. baumannii*. To this end, significant changes in the bacterial structure were observed when the *K. pneumoniae* strain, which is highly resistant to carbapenem and colistin, was exposed to NAC alone or in combination with low concentrations of meropenem. These changes included elongation of the bacteria, disruption of cell integrity, breakdown of the outer cell wall or inner membrane, and forming outer membrane vesicles (OMVs) (De Angelis et al., 2022). Another study also indicated that NAC showed strong antibacterial effects against CR *K. pneumoniae* and CR *A. baumannii* in a way that depended on the concentration. Additionally, NAC showed excellent synergy with both meropenem and ampicillin/sulbactam by restoring their susceptibility (Oliva et al., 2023).

*De Angelis* et al. discovered a strong synergy between NAC and meropenem when used against clinical strains of CR bacteria. Similarly, *Pollini* et al. observed a significant synergistic effect when combining colistin with NAC against CR *A. baumannii* (Pollini et al., 2018a; De Angelis et al., 2022). Additionally, the Ceftazidime/avibactam + NAC combination significantly deteriorated the integrity of bacterial cell membranes (Huang Z. et al., 2023). Therefore, as mentioned, NAC can effectively control CR bacteria and enhance the efficacy of traditional antibiotics against these bacteria. The presence of a NAC thiol group can change the redox state of bacterial periplasm. This change deactivates a controlled mechanism and causes proteins to misfold. These misfolded proteins then build up in the cytoplasm and are released through the creation of OMV (Volgers et al., 2017). Periplasmic bacterial enzymes, including carbapenemase and other beta-lactamases, undergo regulation through a sophisticated regulatory system. In the presence of NAC, these enzymes are deactivated due to protein misfolding, resulting in the loss of their function (De Angelis et al., 2022).

The biofilm community of CR bacteria is also a critical factor for antibiotic resistance in clinical settings. The mucolytic effects of NAC are attributed to its free sulfhydryl group. This group is responsible for breaking the disulfide bonds present in mucus, decreasing its viscosity (Olofsson et al., 2003; Zhao and Liu, 2010; Wendorf et al., 2015). The functional group -SH can disrupt the disulfide bridges of proteins found in the bacteria, causing a loss of their three-dimensional structure and ultimately rendering them inactive. According to these results, it may be inferred that NAC acts by chemically altering the structure of the biofilm and could potentially be a significant agent for combating bacterial biofilms (Samuni et al., 2013; Temel and Erac, 2022). In this regard, *Feng* et al. conducted a study to examine the impact of NAC, both alone and in combination with tigecycline, on *A. baumannii* biofilms. They found that the presence of NAC alone and NAC + tigecycline combinations at low concentrations dramatically reduced the creation of biofilms by the isolates (Feng et al., 2018). In line with these results, another study also reported that NAC + tigecycline combinations could significantly reduce the biofilm formation of CR *A. baumannii* strains (Temel and Erac, 2022). The study also examined the impact of combining NAC and tigecycline on the expression of *A. baumannii* biofilm-related genes, including *bap* (Biofilm associated cell surface protein) and *csuE* (Pilus formation). Following exposure to drug combinations, notable decreases were found in the expressions of the *bap* and *csuE* genes in the strains. The decrease in expression levels may be attributed to the direct impact of the drugs on transcription factors associated with the relevant genes, or it could result from overall inhibition in the quorum-sensing process (January 2017). Therefore, NAC can inhibit the growth of CR bacteria, improve the activity of conventional antibiotics, and destroy the biofilm community of these bacteria. However, data about NAC interaction with CR bacteria are limited, and more confirmatory studies are needed.

## 7 Antimicrobial peptides

Antimicrobial peptides (AMPs) are a group of basic polypeptides consisting of 12–50 amino acid residues. They have significant functions in both innate and adaptive immunity (Takahashi and Yamasaki, 2020; Moghadam et al., 2022). Natural AMPs are found in vertebrates, plants, and small organisms such as bacteria and fungi (Wang et al., 2016). Hence, AMPs are a group of molecules found in the innate immune system that can kill microorganisms and regulate the immune response. They are the initial defense mechanism against invading pathogens (Falanga and Galdiero, 2017).

Studies have shown that AMPs can successfully kill drug-resistant bacteria (Magiorakos et al., 2012; Choi et al., 2021). Noteworthy, AMPs can penetrate or interact with the biofilms created by drug-resistant bacteria, or they can enhance the effectiveness of conventional antibiotics through a synergistic effect (Martinez et al., 2019a). A study showed that combining PapMA-3 (a new PapMA analog) with vancomycin, rifampin, and erythromycin effectively produced synergistic effects against CR *A. baumannii*. Furthermore, PapMA-3 has the potential to enhance the permeability of the bacterium membrane to imipenem and meropenem. PapMA-3 exhibited the ability to inhibit biofilm development at its MIC. Furthermore, it demonstrated the ability to effectively suppress biofilm formation at lower doses when used with antibiotics. PapMA3 disrupted the structure of the bacterial membrane, even at concentrations lower than the MIC (Choi et al., 2021). Besides, the findings of a study on the impact of WAM-1, derived from the mammary gland of the Tammar wallaby, indicate that WAM-1 exhibits potential as a therapeutic agent for treating infections caused by CR *K. pneumoniae*. Furthermore, this AMP also exhibited anti-inflammatory properties (Zhang X. et al., 2022).

Furthermore, P5, a newly created AMP, showed notable synergistic effects when combined with meropenem. It also demonstrated the ability to dissolve biofilms and effectively kill bacteria associated with biofilms, specifically against a CR *P. aeruginosa* (Martinez et al., 2019a). Cec4 was another AMP with an inhibitory effect against CR bacteria. Cec4 is an AMP consisting of 41 amino acids. It has demonstrated inhibitory action against CR *A. baumannii*. Furthermore, Cec4 can remarkably eliminate biofilm formation by this particular bacterium. Significantly, following the administration of Cec4, there were distinct variations in the expression of membrane proteins, bacterial resistance, and pilus-related genes. Cec4 significantly influences the expression of genes that play a role in developing *A. baumannii* biofilms, including *CsuE*, *BfmR*, *BfmS*, *AbaI*, and *Bap* (Liu W. et al., 2020). In the end, WLBU2, a cationic synthetic peptide, indicated good activity against Gram-positive and Gram-negative bacteria (Lin et al., 2018). A study was conducted to explore the antibiofilm impact of WLBU2 against CR *P. aeruginosa*. The results indicated that the WLBU2 peptide exhibits potent inhibitory and eradication effects on the *P. aeruginosa* biofilm. The WLBU2 peptide decreased gene expression levels associated with biofilm growth and maturation (Masihzadeh et al., 2023). Noteworthy, other AMPs with inhibitory effects against CR bacteria are presented in Table 2.

**TABLE 2:** Antibacterial and antibiofilm activity of AMPs against CR bacteria.

Year of publication	AMPs	AMP’s description	Bacteria	Outcome	References
2020	DRGN-6,-7,-8	These peptides are artificially created from the cathelicidin found in Komodo dragons	CR *K. pneumoniae*	These AMPs caused significant increases in the permeability of the cells and considerable depolarization	Hitt et al. (2020)
2019	Cathelicidin-BF15-a4 (ZY4)	The peptide was synthesized by amino acid substitutions based on cathelicidin-BF15	MDR *P. aeruginosa* and *A. baumannii*	ZY4 killed bacteria and persister cells and inhibited the biofilm community. The AMP decreased susceptibility to *P. aeruginosa* lung infection and suppressed dissemination of bacteria to target organs in a mouse septicemia infection model	Mwangi et al. (2019)
2019	CM15 and its ATCUN variants (GGH-CM15 and VIH-CM15)	CM15 is a chimeric peptide from melittin and cecropin-A. ATCUN motifs were designed by adding the tripeptide motifs Gly-GlyHis (GGH) or Val-Ile-His (VIH) to CM15	CR *K. pneumoniae* and *Escherichia coli*	AMPs, when combined with meropenem, streptomycin, or chloramphenicol, showed synergistic effects against biofilms	Agbale et al. (2019)
2017	NN2_0050 and NN2_ 0018	AMPs are designed by LSTM algorithms	MDR *E. coli*, *A. baumannii*, *K. pneumoniae*, *P. aeruginosa*, *Staphylococcus aureus*, and *coagulase-negative staphylococci*	These designed peptides selectively interacted with and disrupted bacterial cell membranes and caused secondary gene regulatory effects	Nagarajan et al. (2018)
2023	GAN-pep 3 and GAN-pep 8	New AMPs generated based on WGAN-GP.	MR *S. aureus* and CR *P. aeruginosa*	Inhibitory effects against both bacteria	Lin et al. (2023)
2022	Epi-1 and hBD-3	Human beta-defensin-3 (hBD-3) is produced by epithelial cells, and Epinecidin-1 (Epi-1) is an AMP derived from the orange-spotted grouper (*Epinephelus coioides*)	CR *K. pneumoniae, Klebsiella aerogenes*, *P. aeruginosa* and *A. baumannii*	Antibacterial activity against all studied clinical isolates. In experimental mouse sepsis models with *K. pneumoniae* and *P. aeruginosa*, increased survival rates were observed with hBD-3 monotherapy, hBD-3 + meropenem, and hBD-3 + Epi-1	Bolatchiev (2022)
2021	LL-37	LL-37 is a synthetic peptide derived from the C-terminal region of the human cationic antimicrobial protein (hCAP)	MDR *E. coli*	Inhibited *mcr-1* carrying, carbapenemase, and ESBL-producing *E. coli*	Morroni et al. (2021)
2022	DGL13K	The D-enantiomers of antimicrobial peptide GL13K, which derived from the salivary protein BPIFA2	CR *K. pneumoniae,* MDR and XDR *P. aeruginosa* and XDR *A. baumannii*	Inhibitory effect against all bacteria	Gorr et al. (2022)
2022	PEP-38 and PEP-137	The new peptides are designed by LSTM RNN.	CR *K. pneumoniae* and *K. aerogenes*	Inhibitory effect against bacteria. PEP-137 showed a survival rate of 50%, while PEP-38 was ineffective in the experimental murine model of *K. pneumoniae*-induced sepsis	Bolatchiev et al. (2022)
2021	MSI-78	The MSI-78, also named pexiganan, is a synthetic analog of maganin-2	CR *K. pneumoniae*	It showed an inhibitory effect, and this antibacterial effect is thought to result from irreversible membrane-disruptive damage	Denardi et al. (2022)
2023	11pep and D −11pep	Two novel antibiotic peptides were designed and synthesized that polymerized the β1, β9, β15, and β16 chains of BamA (BamA, a major component of the outer membrane protein family)	CR *E. coli*, *P. aeruginosa* and MDR *A. baumannii*	Both peptides disrupted the bacterial outer membrane and showed broad-spectrum antibacterial activity. D-11pep effectively inhibited the initial attachment of CR *E. coli* for biofilm formation	Yang et al. (2023)
2024	Osmin	Osmin comprises 17 amino acids and is isolated from solitary bee (*Osmia rufa*) venom	drug-resistant *K. pneumoniae*	Reduced bacterial growth and the expression of pro-inflammatory cytokines and fibrosis-related genes in mice with CR *K. pneumoniae* sepsis	Jeon et al. (2024)
2012	LLKKLLKKC ((LLKK)_2_C) and CLLKKLLKKC (C(LLKK)_2_C)	The cationic amphiphilic alpha-helical peptides	CR *A. baumannii*	These peptides showed excellent potency in mouse models of peritonitis and pneumonia infections caused by CR *A. baumannii*	Huang et al. (2012)
2021	LyeTx I-b and PEGylated LyeTx I-b (LyeTx I-bPEG)	LyeTx I-b is a synthetic peptide derived from native LyeTx I, originally isolated from *Lycosa erythrognatha* spider venom	CR *A. baumannii*	LyeTx I-b was active against *A. baumannii*. LyeTx I-bPEG, was slightly less active than its analogue. PEGylation improved the anti-biofilm activity of LyeTx I-b	César Moreira Brito et al. (2021)
2021	1B and C	Two derivatives of the Temporin L from *Rana temporaria*	CR *K. pneumoniae*	Both peptides were able to inhibit the growth of carbapenemase-producing strains effectively	Roscetto et al. (2021)
2018	PaDBS1R1	It is a novel cationic antimicrobial peptide engineered by ribosomal protein L39E from the hyperthermophilic archaeon *Pyrobaculum aerophilum*	CR *K. pneumoniae*	Induced permeabilization and depolarization of the cytoplasmic bacterial membrane, leading to leakage of the intracellular content and finally cell death	Irazazabal et al. (2019)
2020	sDq-3162	It’s a 28-residue ponericin G-like dinoponeratoxin from the giant ant *Dinoponera quadriceps* venom	CR bacteria (i.e., *A. baumannii*, *K. pneumoniae, P. aeruginosa* and *E. coli*)	Displayed a significant bacteriostatic and bactericidal effect	Dodou Lima et al. (2020)
2024	AS-12W	Cathelicidin AS-12W Derived from the *Alligator sinensis*	CR *P. aeruginosa*	Demonstrated broad-spectrum antibacterial activity and removed CR *P. aeruginosa* from blood and organs. This peptide could neutralize the negative charge on the surface of the bacteria and disrupt the integrity of the bacterial cell membrane. The peptide can bind to the genomic DNA of bacteria and stimulate the production of ROS within bacteria	Zhang et al. (2024)
2017	AM-CATH36, AM-CATH28, AM-CATH21	These peptides are cathelcidin and two shorter fragments from *Alligator mississippiensis* (American alligator)	MDR *A. baumannii* and CR *K. pneumoniae*	Strong activity against bacteria. These peptides permeabilize the bacterial membrane	Barksdale et al. (2017)
2024	Gy-CATH	A novel anionic antimicrobial peptide identified from the skin of the frog *Glyphoglossus yunnanensis*	*CR E. coli*	Preventive and therapeutic capacities in mice that are infected with bacteria	He et al. (2024)
2021	Octopromycin	A novel peptide derived from *Octopus minor*	MDR *A. baumannii*	Increased ROS production inhibited the biofilm formation and showed biofilm eradication activity. *In vivo* study results revealed that the *A. baumannii*-infected fish treated with this peptide exhibited a significantly higher relative percent survival (37.5%) than the infected mock-treated fish with PBS (16.6%)	Rajapaksha et al. (2021)
2015	Tilapia piscidin 3 (TP3) and tilapia piscidin 4 (TP4)	Synthetic antimicrobial peptides from an aquatic organism *Oreochromis niloticus*	MDR *A. baumannii* and CR *K. pneumoniae*	Showed antibacterial effects and administration of these peptides 30 min after infection with bacteria significantly increased survival in mice	Pan et al. (2015)
2022	RaCa-1, RaCa-2, RaCa-3 and RaCa-7	Novel AMPs were identified using amplify derived from the *Rana [Lithobates] catesbeiana* genome	CR *E. coli*	These peptides were active against CR strain with MIC = 2–44 μM	Li et al. (2022b)
2018	IARR-Anal10	The analog derived from the antimicrobial peptide mBjAMP1 isolated from *Branchiostoma japonicum*	MDR *K. pneumoniae*	Suppressed the virulence of *K. pneumoniae* to a degree similar to tigecycline and did not induce development of resistance by this bacterium	Park et al. (2018)
2019	Pen-BR, Pen-RRR and Cecropin P1 (CECP1), Cap11-1–18 m^2^ (CapM2)	Pen-BR, Pen-RRR were generated by fusing HEXIM1 BR and BR-RRR12 peptides with a cell-penetrating peptide, Pen. CECP1 is an AMP from *Ascaris suum*. CapM2 is a derivative of guinea pig cathelicidin CAP11	CR *E. coli* and *P. aeruginosa*	Showed improved and potent bacterial inhibitory and killing activities	Ho et al. (2019)

AMPs: antimicrobial peptides. ATCUN: amino terminal Cu(II) and Ni(II). CR: carbapenem-resistant. LSTM: long short-term memory. MR: Methicillin-resistant. MDR: multidrug-resistant. PBS: phosphate-buffered saline. ROS: reactive oxygen species. RNN: recurrent neural network. XDR: extensively drug-resistant. WGAN-GP: Wasserstein generative adversarial network with gradient penalty.

As mentioned, AMPs can decrease the expression of genes related to biofilms, hinder the initial attachment of bacteria to a surface, target bacteria before they can form a biofilm, eliminate bacteria already embedded in biofilms, or eradicate developed biofilms. These actions effectively inhibit or eliminate biofilms (Liu W. et al., 2020). Additionally, they demonstrated promise when combined with conventional antibiotics in combination therapy. AMPs can be readily modified by substituting their amino acid residues to combat drug resistance, thereby creating new and highly effective AMPs (Choi et al., 2021).

AMPs can be categorized into two primary groups based on their mechanisms: (1) direct eradication by altering the integrity of the cell membrane or affecting the production of internal components such as nucleic acids and proteins, and (2) regulating the immune response to eliminate harmful infections (Beaumont et al., 2014; Yang M. et al., 2019). In addition, AMPs exhibit several additional activities, such as inducing membrane depolarization and destabilization (Irazazabal et al., 2019; Hitt et al., 2020). AMPs can also induce cell apoptosis by regulating the production of ROS (Hwang et al., 2011). Besides, AMPs work as immunomodulators by attracting and stimulating immune cells such as neutrophils, mast cells, macrophages, and T cells. Consequently, this affects the roles of neutrophils in generating chemokines (Di Nardo et al., 2008; Pundir et al., 2014).

Therefore, unlike empirical antibiotics that target single or specific bacterial processes, AMPs exhibit multifunctional bacterial killing effects (Hurdle et al., 2011; Gee et al., 2013). To this end, the likelihood of antimicrobial peptides developing resistance is low, as microorganisms would need to undergo substantial changes to their gene sequences, membrane structure, and lipid composition to evade the peptides (Zasloff, 2002; Hein-Kristensen et al., 2013; Ravensdale et al., 2016). For this reason, due to their wide-ranging effectiveness and minimal harm to the host, antimicrobial peptides have garnered increased interest as potential therapeutic agents against drug-resistant bacteria (Ho et al., 2019).

Collectively, AMPs show promise in controlling MDR bacteria and associated biofilm communities. However, these biomacromolecules have some limitations, including their susceptibility to degradation, limited absorption, distribution, metabolism, and excretion (ADME) capabilities, transport mechanism, target delivery, and potential toxicity (Di, 2015; Khara et al., 2015; Wagner et al., 2018; Wang et al., 2019). To this end, recently published studies have been conducted to enhance the therapeutic effectiveness of these biomolecules and mitigate their negative side effects. There are two methods for this objective. One approach involves modifying peptides that have already been altered to enhance their effectiveness against pathogens. This modified peptide can be achieved by changing, removing, adding, or substituting amino acids in the original sequence. Additionally, modifications can be made to the N or C terminal parts of the peptides, such as cyclizing or conjugating them with antibiotics or other molecules (Radzishevsky et al., 2007; Costa et al., 2015; Liu et al., 2017). Another method entails utilizing nanotechnology via nanoparticles to inhibit peptide breakdown, enhance antimicrobial effectiveness and bioavailability, amplify selectivity, and regulate the administered dosage according to physicochemical factors such as time, temperature, and pH (Roque-Borda et al., 2022). Therefore, AMPs showed potential for managing CR bacteria; however, some drawbacks limited their clinical usage. In this regard, scientists should consider using the abovementioned approaches to improve AMPs’ performance against CR bacteria.

## 8 Conclusion

Antibiotic resistance and MDR bacteria are challenging and threatening to the global community. One of the main reasons is the indiscriminate use of antibiotics, which causes the creation of new antibiotic-resistant strains at a high rate. One of the most important causes of death in the world is infections caused by antibiotic-resistant bacteria. Therefore, the synthesis of new and effective antimicrobial agents is critical. Evidence from scientific investigations indicates that the progress in creating antibiotics is not keeping pace with the rise of antibacterial resistance patterns, particularly for significant bacterial infections. Multiple antibacterial resistance profiles, such as the CR bacterium, have been recently identified. Recent studies have documented the potential of phages, nanoparticles (drug platforms), and natural substances for managing these bacteria. Moreover, various management approaches have been employed to address the issue of resistant pathogens. These include (a) gaining a thorough understanding of resistance at the molecular level, as well as its evolution and spread; (b) discovering novel chemical agents with antibiotic properties; and (c) improving the effectiveness of antibiotics through innovative methods like combination therapy. It is noteworthy to mention that there is a growing prevalence of bacteria that are becoming increasingly resistant. Hence, it is imperative to employ other strategies to manage resistant infections, given that antibiotic resistance poses a significant challenge in clinical environments. However, there is a lack of extensive clinical data and *in vitro* studies in this area. Therefore, further research is needed to determine the most effective non-antibiotic approaches that cause minimal harm to humans, enhance their impact on bacterial pathogens, identify the optimal timing for treatment, and establish the appropriate route and administration dosage. However, non-antibiotic methods could soon be implemented as a viable antibiotic substitute.
